# An Account of the Practice of One of the Physicians of the Westminster General Dispensary, and of the Western Dispensary, from the 20th of May, to the 20th of June, 1807

**Published:** 1807-07

**Authors:** Samuel Fothergill

**Affiliations:** Southampton Street, Strand


*An Account of the Practice of one of the Physicians of the Westminster General Dispensary, and of the Western Dispensary, from the 20th of May, to the 20th of June,* 1807.

| | | | |
|---|---|---|---|
| ACUTE DISEASES. | | Hypochondriasis | 3 |
| Synochus | 8 | Gout | 1 |
| Typhus | 6 | Gastrodynia | 7 |
| Tertian | 2 | Diarrhœa | 4 |
| Rheumatism | 4 | Dyspepsia | 6 |
| Pleurisy | 2 | Palpitation | 2 |
| Catarrh | 7 | Colic | 4 |
| Puerperal Fever | 1 | Jaundice | 2 |
| Small-pox | 1 | Constipation & Vomiting | 3 |
| Measles | 3 | Dysentery | 2 |
| Hooping Cough | 4 | Hæmorrhoids | 2 |
| Acute Diseases of Infants | 15 | Hæmatemesis | 1 |
| CHRONIC DISEASES. | | Hæmoptoe | 3 |
| Asthenia | 18 | Worms | 5 |
| Pulmonary Consumption | 4 | Tumid Abdomen | 3 |
| Marasmus | 2 | Scurvy | 2 |
| Cough and Dyspnœa | 20 | Cutaneous Diseases | 8 |
| Chronic Catarrh | 5 | Dropsy | 5 |
| Pleurodyne | 2 | St. Vitus's Dance | 1 |
| Chronic Rheumatism | 6 | Epilepsy | 2 |
| Hemicranium | 2 | Chlorosis | 3 |
| Lumbago | 3 | Dysmenorrhœa | 2 |
| Cephalalgia | 4 | Amenorrhœa | 4 |
| Paralysis | 3 | Menorrhagia | 3 |

Coughs and pulmonic affections, which at the latter end of the last, and the beginning of the present month, were both frequent and troublesome, are now declining; and seem to yield more easily to the medicines employed. As they are in a great degree occasioned by the variations of weather, so when that becomes settled, and the temperature of the air is milder, these complaints gradually terminate. Another description of diseases now occurs, not so frequent or so general, but more formidable and alarming; ing; the cases of fever which I have yet observed, have been, however, less severe than usual; some of them have already terminated favourably, and the rest appear to be going on well.

Some medical practitioners deny that the plague, the yellow fever, and typhus are contagious diseases; happily, in this country, we know little of the two first, except by report; in the latter, the most ample experience and collection of facts, sufficiently prove its contagious nature. We cannot, it is true, in every case ascertain that the complaint originated from communication with diseased persons; nor will the actual communication always produce fever; many predisposing causes are requisite; and the human constitution is evidently less susceptible of disease at one time than another. In the present Report, the first cases of typhus occurred in a woman, who had been exposed to considerable fatigue, in which state she was wet through, and wore her damp clothes during the day; she felt very chilly, and the usual symptoms of fever soon after came on. She lived in a close room; and her husband, who worked in the same apartment, in about ten days became also affected with fever; when I saw them, it had continued nearly three weeks, and was evidently typhoid; they had lain together in the same bed; and as the man was previously in a sound state of health, and knew of no probable cause for his illness, the inference would naturally be, that he had received it from his wife. Whoever has observed the symptoms of typhus, might readily suppose that the surrounding atmosphere, to an extent more or less great, particularly in a small close room, might become sufficiently impregnated with the particles continually exhaling from the diseased body, to infect others with a similar disease. Under certain circumstances, any fever whatever will become typhoid; and foul air, and putrid noxious exhalations may at any time generate it. Thus we find typhus fever breaking out in jails, in badly regulated hospitals, in camps, in prison-ships; and wherever a number of people are crowded together in a small place not properly ventilated; and any sound healthy person, remaining in such a place a sufficient length of time, varying in different people, will become affected.

Three of the other cases were in one family; the mother, a widow, had been in the country, and returning with her youngest child, found her eldest girl, aged 10 years, ill of a fever, which had continued about two weeks. The mother and the infant slept with this child, and they were both both in a few days, seized with the symptoms of typhus; from which they are now recovering. The treatment which I have found most successful, has been, to keep the bowels open with small doses of calomel, repeated every two or three days; when the skin is very hot and dry, saline mixture and antimonial wine; and when this is not the case, bark and cordials; and wine where it does not increase the heat; opiates occasionally; and where the head is much affected, shaving it, blistering, and sometimes cupping, seem to afford relief. Amongst the class of patients which we generally attend at Dispensaries, it is extremely difficult to introduce cold affusion; the physician cannot always stay to see it done, and it is not safe to trust people indiscriminately with the administration of a remedy, highly potent and beneficial, but which, misapplied, would often be attended with consequences fatal to the patient, and prejudicial to a practice, the promoters of which merit immortal honour.

SAMUEL FOTHERGILL.

*Southampton Street, Strand, June 25, 1807.*

